# Alleviation of allergic rhinitis symptoms in an animal model by *Lactiplantibacillus plantarum* BGI-N6

**DOI:** 10.3389/fimmu.2026.1923543

**Published:** 2026-08-19

**Authors:** Sihang Cai, Xiaowei Xu, Xiaofan Sun, Qiang Luo, Weijian Chen, Xihe Wang, Jielei Zhu, Yanhong Liu, Liang Xiao, Haifeng Zhang, Yuanqiang Zou, Yiyi Zhong

**Affiliations:** 1 State Key Laboratory of Genome and Multi-omics Technologies, BGI Research, Shenzhen, China; 2 College of Life Sciences, University of Chinese Academy of Sciences, Beijing, China; 3 BGI Precision Nutrition, Shenzhen, China; 4 BGI Research, Shenzhen, China; 5 Shenzhen Key Laboratory of Human Commensal Microorganisms and Health Research, BGI Research, Shenzhen, China

**Keywords:** allergic rhinitis, gut microbiota, lactiplantibacillus plantarum, metagenomics, probiotics

## Abstract

Allergic rhinitis (AR) is a chronic inflammatory disease with rising global prevalence and a substantial public health burden. Current treatments have limited efficacy and tolerability, highlighting the need for new strategies. Probiotics represent a promising approach due to their ability to modulate gut microbiota and host immunity. Here, we investigated the preventive potential of *Lactiplantibacillus plantarum* BGI-N6 in an OVA/ALUM-induced AR rat model. BGI-N6 administration alleviated AR symptoms and nasal mucosal pathology, reduced key allergic mediators, shifted serum immunoglobulin and cytokine levels toward normal, and restored the Th1/Th2/Th17/Treg balance. Metagenomic sequencing of cecal contents showed that these effects were accompanied by expansion of Bacteroidota-affiliated SCFA-producing taxa, restoration of microbial functional capacity, and identification of 41 core functional genes (KEGG Orthologues) consistently shifted across all three dose groups, with Bacteroides showing the strongest enrichment. Correlation analyses further connected these microbial shifts with immune parameters. These findings support BGI-N6 as a probiotic intervention for AR and implicate gut microbiota remodeling as a central correlate of probiotic-induced immunomodulation.

## 1 Introduction

Allergic rhinitis (AR) is a chronic IgE-mediated inflammatory disease that affects up to 50% of the population in some high-income countries, and its prevalence is steadily increasing in low- and middle-income countries (1). It is particularly common among children and adolescents: the International Study of Asthma and Allergies in Childhood (ISAAC) reported a worldwide prevalence of 14.6% in 13–14 year olds, and 80% of patients develop symptoms before age 20 (2). The resulting impairments in sleep, school performance, and work productivity make AR a substantial public health burden (3). The disease arises from environmental exposures acting on a predisposed genetic background, with established risk factors including air pollutants (diesel exhaust particles), maternal and paternal smoking, heavy maternal smoking during the first year of life, indoor allergens (house dust mites and animal dander), and early-life factors such as antibiotic use and reduced exposure to infectious agents (**1**,^3^). AR pathogenesis involves two phases: sensitization and elicitation. During initial allergen exposure, dendritic cells present processed antigens to naïve CD4^+^ T cells and drive their differentiation into Th2 cells. Th2 cells secrete cytokines IL-4 and IL-13, which promote B cell class switching and subsequent IgE production. The synthesized IgE binds to high-affinity receptors on the surface of mast cells, sensitizing them. Upon re-exposure, the allergen cross-links mast cell-bound IgE. Mast cells then degranulate and release inflammatory mediators including histamine, prostaglandins, and leukotrienes. Consequently, patients develop rhinorrhea, nasal congestion, sneezing, and pruritus (1).

**Figure 1:**
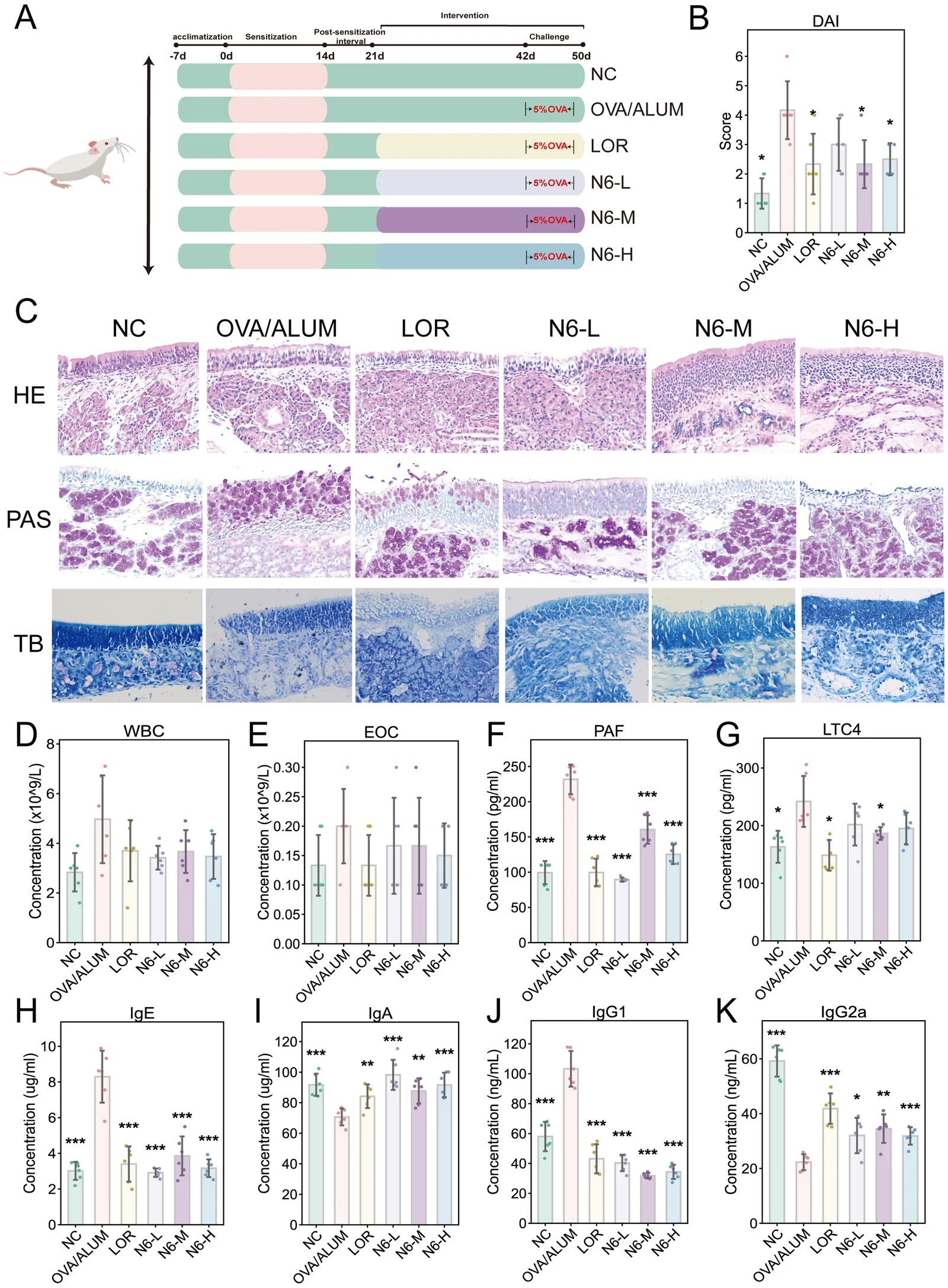
Effects of BGI-N6 intervention on AR symptoms and nasal mucosa morphology. **(A)** AR Rat Experiment Procedure. **(B)** Rat DAI Index Score. **(C)** Representative Images (400X) of Rat Nasal Mucosal Tissue Stained with HE, PAS, and TB. **(D, E)** White blood cell and eosinophil levels in blood. **(F–K)** Serum levels of PAF, LTC4, IgE, IgA,IgG1 and IgG2a. Data are presented as mean ± standard deviation (SD); *p < 0.05, **p < 0.01, ***p < 0.001. Each dot corresponds to an individual animal (n = 6 per group).

Current treatments for AR include environmental control, pharmacotherapy, and allergen-specific immunotherapy (AIT). None of them offers a fully satisfactory solution. Complete allergen avoidance is hard to achieve. Pharmacological agents such as antihistamines and corticosteroids offer symptomatic relief but are associated with adverse effects, including sedation, xerostomia, and endocrine disturbances with prolonged use (4). AIT, the only treatment capable of modifying the natural course of allergic disease, induces incremental immunological tolerance through stepwise escalation of allergen dosing (5); however, it requires a 3–5 year course of regular clinic visits or daily self-administration that limits patient adherence, is constrained by high cost and the risk of severe systemic reactions, and is estimated to be used in fewer than 10% of eligible patients globally (5, 6). Therefore, there is an urgent imperative to develop novel and more effective immune intervention strategies.

The gut microbiota plays a fundamental role in host immunity (7): supporting the development and maturation of the immune system (8), directing immune cell differentiation (9), and promoting the production of immune mediators (10, 11). Beyond immune regulation, the gut microbiota contributes to metabolic pathways, generating critical metabolites such as short-chain fatty acids (12), amino acids (13), vitamins (14), and bile acids (15), all of which collectively maintain host immune homeostasis. Dysbiosis of the gut microbiota has been closely linked to AR (16–18): patients exhibit reduced microbial diversity and altered community composition, characterized by decreased relative abundance of Bacteroidetes, and studies in high-prevalence allergy regions have consistently reported lower *Lactobacillus*, higher *Staphylococcus* and *Clostridium* in the gut microbiota of affected children (19, 20). The gut-nose axis provides a mechanistic basis for this link—microbially derived metabolites, particularly SCFAs, enter the systemic circulation and modulate Th2/Treg balance at distant mucosal sites, including the nasal epithelium (21). Given this interplay, probiotics have attracted significant research interest for their potential to modulate the gut milieu and host immunity, with specific strains showing potential in alleviating AR symptoms (22).

Although these early results are encouraging, probiotic effects are highly strain-dependent, and individual studies vary considerably in the strains tested, intervention duration, and outcome measures, making it difficult to generalize findings or formulate consistent clinical recommendations for AR (23). *L. plantarum* has been widely studied as a probiotic, and several strains have shown potential in alleviating AR (24–32). However, many studies have used mixed probiotic formulations that may obscure the contribution of individual strains. In this study, we used an OVA/ALUM-induced AR rat model and metagenomic sequencing to investigate the immunomodulatory ability of *L. plantarum* BGI-N6, a single strain isolated from healthy volunteers with a documented safety profile (33). Our findings provide preclinical evidence for BGI-N6 as a strain-specific probiotic candidate for AR.

## 2 Materials and methods

### 2.1 Bacterial strain and preparation

The *L. plantarum* BGI-N6 was obtained as a lyophilized powder (5 × 10¹¹ CFU/g) from BGI Precision Nutrition (Shenzhen) Technology Co., Ltd. To prepare the administration, the powder was suspended in sterile physiological saline and adjusted to the target doses for each specific group. The animals were then dosed with this probiotic suspension by oral gavage.

### 2.2 Experimental design and grouping of animals

Eight-week-old female Sprague-Dawley rats (SPF grade, 200 ± 20 g) were randomly assigned to six groups (n = 6 per group) and acclimatized for 7 days under barrier conditions with ad libitum access to standard maintenance diet (MD17121, Medicience, Jiangsu, China) and water. A sample size of six per group was adopted, consistent with standard practice in probiotic intervention studies. The experimental protocol comprised four phases: sensitization, post-sensitization interval, intervention, and challenge (**Figure 1**; Approval No. BGI-IRB A24040).

During the sensitization phase (days 1–13), all groups except the normal control (NC) group received intraperitoneal injections of 1 mL sterile saline containing 0.3 mg ovalbumin (OVA; Grade II, batch A5253, Sigma-Aldrich, USA) and 30 mg aluminum hydroxide adjuvant (batch 239186, Merck, USA) every other day, for a total of seven injections; the NC group received an equal volume of sterile saline. The OVA preparation was research-grade and not certified for endotoxin levels.

During the post-sensitization interval (days 14–21), all animals were maintained under standard housing conditions without any intervention, allowing systemic immune sensitization to stabilize before the initiation of probiotic administration.

During the intervention phase (days 22–50), rats received daily oral gavage (1 mL) for 28 consecutive days as follows: the NC and OVA/ALUM groups received sterile saline; the BGI-N6 low-, medium-, and high-dose groups (N6-L, N6-M, and N6-H) received bacterial suspensions at 1 × 10^7^, 1 × 10^8^, and 1 × 10^9^ CFU/mL in saline, respectively; and the positive control group (LOR) received loratadine at 0.9 mg kg^-1^ body weight (human-equivalent dose based on 10 mg/60 kg).

During the challenge phase(days 42-50), all groups except the NC group received intranasal instillation of 0.1 mL 5% (w/v) OVA solution per nostril on days 42, 44, 46, 48 and 50 (every other day, 2 h after the daily gavage); the NC group received an equal volume of sterile saline. AR symptoms (sneezing, nasal scratching, and rhinorrhea) were scored over a 10-min period immediately following the final challenge on day 50, after which animals were terminally anesthetized. Blood was collected via the abdominal aorta, and the spleen, thymus, nasal mucosal tissue, and cecal contents were harvested and stored at −80 °C.

### 2.3 DAI Score

The Disease Activity Index (DAI) was used to quantitatively assess AR symptom severity based on three cardinal symptoms: sneezing, nasal scratching/rubbing, and rhinorrhea. Each parameter was graded on a 4-point scale (0 to 3). Specifically, sneezing was scored based on frequency (0: absent; 1: ≤3 episodes; 2: 4–10 episodes; 3: ≥11 episodes). Nasal scratching was rated from 0 (none) to 3 (vigorous rubbing), with scores of 1 and 2 indicating mild and frequent scratching, respectively. Rhinorrhea was evaluated as 0 (none), 1 (discharge visible at nostrils), 2 (discharge spilling beyond nostrils), or 3 (discharge spreading across the face). The final DAI was obtained by summing the three sub-scores. Because the DAI is an ordinal composite score (range 0–9), between-group differences were assessed using the Kruskal–Wallis test followed by pairwise Mann–Whitney U tests with Benjamini–Hochberg correction.

### 2.4 Nasal mucosal tissue section staining

After fixation in 4% paraformaldehyde for 24 hours, the nasal mucosa samples were processed through a standard graded ethanol series, cleared with xylene, and embedded in paraffin. Tissue sections were first evaluated for overall histopathology and epithelial damage using hematoxylin and eosin (HE) staining. Furthermore, Periodic acid-Schiff (PAS) and toluidine blue (TB) stains were applied to visualize goblet cells and assess mast cell infiltration, respectively.

### 2.5 Blood biochemical and serum factor analysis

Whole blood was collected from the abdominal aorta into EDTA-anticoagulant tubes, and the numbers of white blood cells (WBC) and eosinophils (EOS) were determined using an automated hematology analyzer. Serum was separated by centrifuging at 1,100 × g for 30 min at 4 °C. Serum levels of IgE, IgA, IgG1, IgG2a, PAF, LTC4, IFN-γ, TNF-α, IL-2, IL-4, IL-5, IL-6, IL-10, IL-12, IL-13, IL-17, and TGF-β were measured using commercially available ELISA kits (Jiangsu Meimian Industrial Co., Ltd., China) according to the manufacturer’s instructions.

### 2.6 Flow cytometry analysis of splenic T lymphocytes

Splenic single-cell suspensions were prepared and stimulated with Cell Activation Cocktail (BioLegend) for 6 h prior to intracellular cytokine staining for Th subset analysis; unstimulated cells were used for Treg detection. Dead cells were excluded using Fixable Viability Stain 780 (BD Biosciences). For surface staining, cells were incubated with anti-CD4-PerCP/Cy5.5 and anti-CD25-AF488 (BioLegend), followed by fixation and permeabilization using an appropriate fixation/permeabilisation kit. Intracellular targets were detected using anti-Foxp3-APC (Thermo Fisher Scientific) for Tregs, and anti-IFN-γ-FITC (BD Biosciences), anti-IL-4-PE (BioLegend), and anti-IL-17A-PE-Cy7 (Thermo Fisher Scientific) for Th1, Th2, and Th17 subsets, respectively. Data were acquired on a flow cytometer and analyzed using FlowJo software.

### 2.7 DNA extraction and metagenomics sequencing

Caecal contents were collected and immediately stored at −80 °C until processing. Metagenomic DNA was extracted using the MagPure Stool DNA KF Kit B (MAGEN, China) according to the manufacturer’s instructions. The extracted DNA was aliquoted into DNase-free tubes (Axygen, USA) and stored at −20 °C until further use.

For metagenomic sequencing, qualified DNA was used to construct libraries with the MGIEasy Universal DNA Library Prep Set. Sequencing was performed on the DNBSEQ-T10 platform (BGI, Shenzhen, China) in a 100 bp paired-end mode. Raw sequencing reads were subjected to quality control and adapter trimming using Fastp (v0.23.4) with the following parameters: --length-required 70, --adapter_sequence AAGTCGGAGGCCAAGCGGTCTTAGGAAGACAA, --adapter_sequence_r2 AAGTCGGATCGTAGCCATGTCGTTCTGTGAGCCAAGGAGTTG.CNP0009371.

### 2.8 Integrated metagenomic & functional analysis of the gut microbiota

Taxonomic classification of quality-filtered reads was performed using Kraken2 (v2.2.14). Beta-diversity was assessed using species-level Bray–Curtis dissimilarity and visualized by principal coordinate analysis (PCoA). Between-group differences in community composition were tested by PERMANOVA (999 permutations, adonis2, vegan, R) with group as the sole factor; homogeneity of group dispersions was confirmed by betadisper (p > 0.05). To identify higher-level taxonomic shifts across all six groups, LEfSe was employed and visualized as a cladogram (LDA > 3.7). To identify differentially abundant species in pairwise comparisons against the OVA/ALUM group, LEfSe was applied at LDA > 3.0 (LDA > 2.0 for NC vs. OVA/ALUM). All analyses used p < 0.05, with thresholds validated by sensitivity analysis across LDA 2.0–4.0 (**Supplementary Table 1**). Functional profiles were generated with HUMAnN3 (v3.0) using the ChocoPhlAn nucleotide database and UniRef90 protein database; features were regrouped into KEGG Orthology (KO) identifiers and mapped to KEGG pathway maps to calculate pathway abundance. Genus-level taxonomic contributions to individual KOs were extracted from HUMAnN3 stratified output to link functional features with their putative taxonomic origins. Pathway-level enrichment was assessed using ReporterScore, with |ReporterScore| ≥ 2.5 considered significantly enriched. Differential KO abundance was analyzed with DESeq2 (v1.48.2) at Benjamini–Hochberg adjusted p < 0.05. Spearman’s rank correlation was used to examine associations among microbial genera, core KO abundances, and clinical phenotypic parameters, with all correlation p-values Benjamini–Hochberg adjusted.

### 2.9 Statistical analysis

Data are presented as mean ± SD. Statistical analyses were performed in Python (v3.13.2) and R (v4.5.1). For continuous variables, group differences were assessed by one-way ANOVA with Welch’s t-test for pairwise comparisons. For the DAI, an ordinal composite score, the Kruskal–Wallis test and Mann–Whitney U tests with Benjamini–Hochberg correction were used. All p-values from multiple pairwise comparisons and Spearman correlation analyses were adjusted using the Benjamini–Hochberg procedure. Significance was defined as p < 0.05. Figures were generated using ggplot2 (v3.5.0) in R and matplotlib in Python.

## 3 Results

### 3.1 BGI-N6 intervention alleviates allergic symptoms in AR rats

To ensure a robust AR phenotype for intervention evaluation, female rats were used in this study because estrogen enhances Th2-biased allergic responses (34, 35). Evaluation of clinical symptoms indicated that the DAI score of the OVA/ALUM group was significantly higher than those of the NC (**Figure 1**). Furthermore, histological examination of the nasal mucosa (**Figure 1**) confirmed severe structural and inflammatory changes in the OVA/ALUM-challenged rats. Compared to the NC group, these animals displayed pronounced inflammatory cell infiltration, damaged epithelial ciliary architecture, and partial glandular hyperplasia. Special staining further highlighted a smoothly thickened basement membrane (PAS) alongside extensive mast cell accumulation and active degranulation (TB). Collectively, these findings confirm the successful establishment of the AR rat model following repeated OVA/ALUM exposure.

Compared to the AR model group, nasal mucosal injury was markedly attenuated in the LOR and all BGI-N6-treated groups, as evidenced by restored epithelial integrity, suppressed glandular hyperplasia and vacuolar degeneration, alongside reduced infiltration of goblet cells and mast cells. Concurrently, overall levels of WBC and EOS displayed a consistent downward trend following BGI-N6 administration(**Figure 1D**). Serum antibody and lipid mediator profiling revealed that the OVA/ALUM challenge increased the levels of PAF, LTC4, IgE, and IgG1, accompanied by decreased IgA and IgG2a, indicating an immune response associated with AR. BGI-N6 administration effectively reversed these alterations, substantially suppressing the OVA-induced elevations of PAF, LTC4, IgE, and IgG1, while promoting IgA and IgG2a levels (**Figure 1F**). Together, these results indicate that BGI-N6 effectively ameliorates allergic symptoms, reduces nasal mucosal inflammation, and restores systemic immune balance in AR rats.

### 3.2 BGI-N6 intervention modulates T lymphocyte subpopulation ratios and immune-related cytokines in AR rats

**Figure 2:**
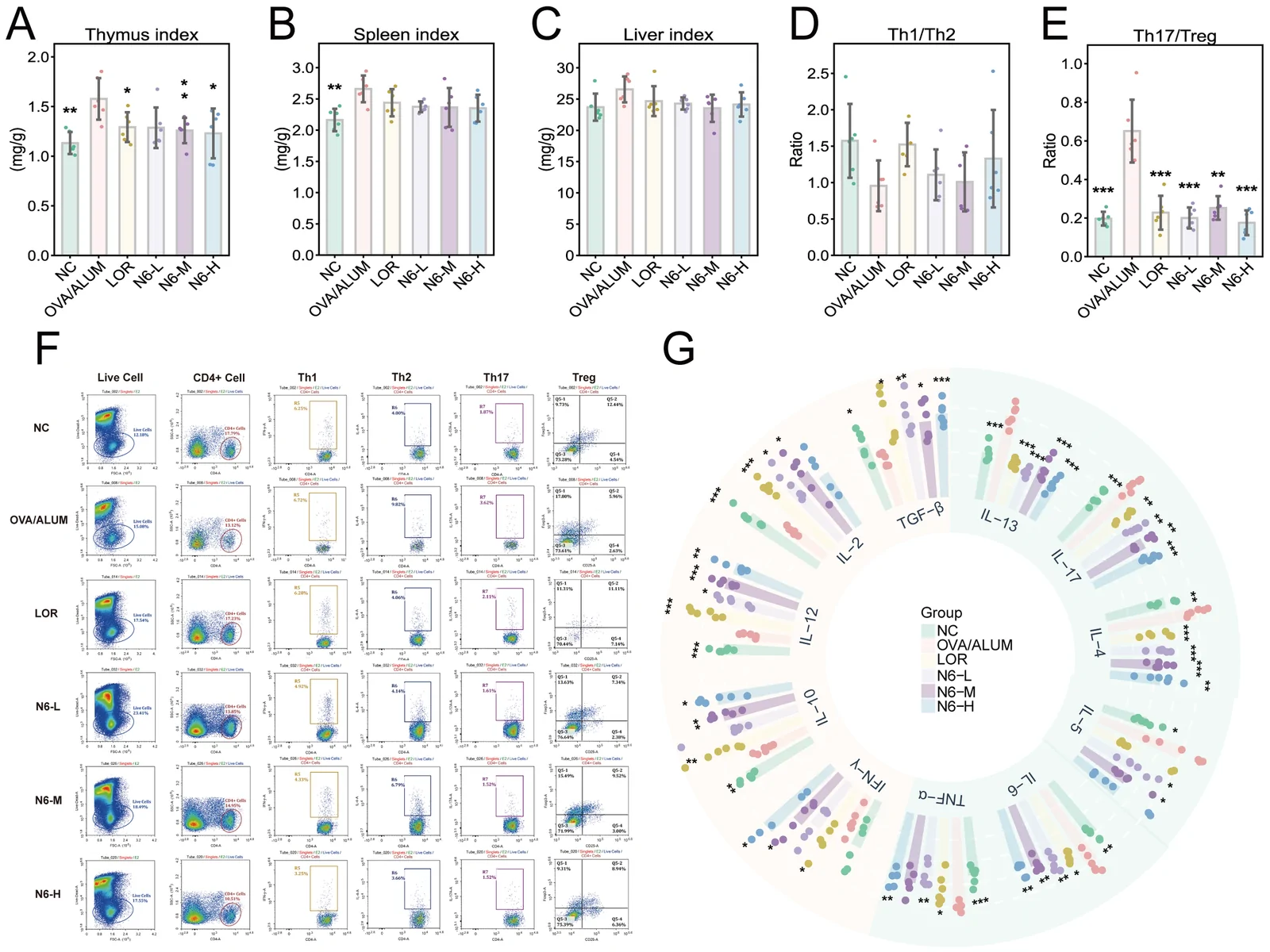
Effects of BGI-N6 on T cell subsets, cytokines, and immune markers in AR rats. **(A, B)** Thymus and Spleen indices. **(C, D)** The ratios of Th1/Th2 and Th17/Treg cells in the spleen. **(E)** Representative flow cytometry plots of splenic T lymphocyte subsets. **(F)** Serum levels of inflammatory cytokines. **(G)** Circular bar chart summarizing cytokine changes across groups. Bar height represents the normalized mean relative to OVA/ALUM. Background shading indicates the overall trend (blue, decreased; orange, increased). Data are presented as mean ± standard deviation (SD); *p < 0.05, **p < 0.01, ***p < 0.001. Each dot corresponds to an individual animal (n = 6 per group).

Assessment of immune organ indices showed that the thymus and spleen indices were significantly elevated in the OVA/ALUM group compared with the NC group (**Figure 2A**), indicative of systemic immune hyperactivation. In contrast, BGI-N6 administration significantly attenuated these OVA/ALUM-induced increases. Flow cytometric analysis demonstrated that BGI-N6 administration improved the Th1/Th2 ratio and significantly reduced the elevated Th17/Treg ratio in the AR model, which was achieved by promoting Treg differentiation and concurrently suppressing the Th17 cell population (**Figure 2C**). Cytokine profiling further revealed that BGI-N6 administration markedly decreased the levels of Th2-associated cytokines (IL-4, IL-13), the Th17-related cytokine IL-17, and pro-inflammatory mediators (TNF-α, IL-6), while significantly increasing the levels of Th1-associated cytokines (IL-12) and Treg-related cytokines (TGF-β) (**Figure 2**; **Supplementary Figures 1A–K**). Collectively, BGI-N6 administration was associated with restoration of the balance among Th1/Th2/Th17/Treg cell subsets and consequently rebalancing the systemic cytokine profile.

### 3.3 BGI-N6 intervention alters the gut microbial composition in AR rats

**Figure 3:**
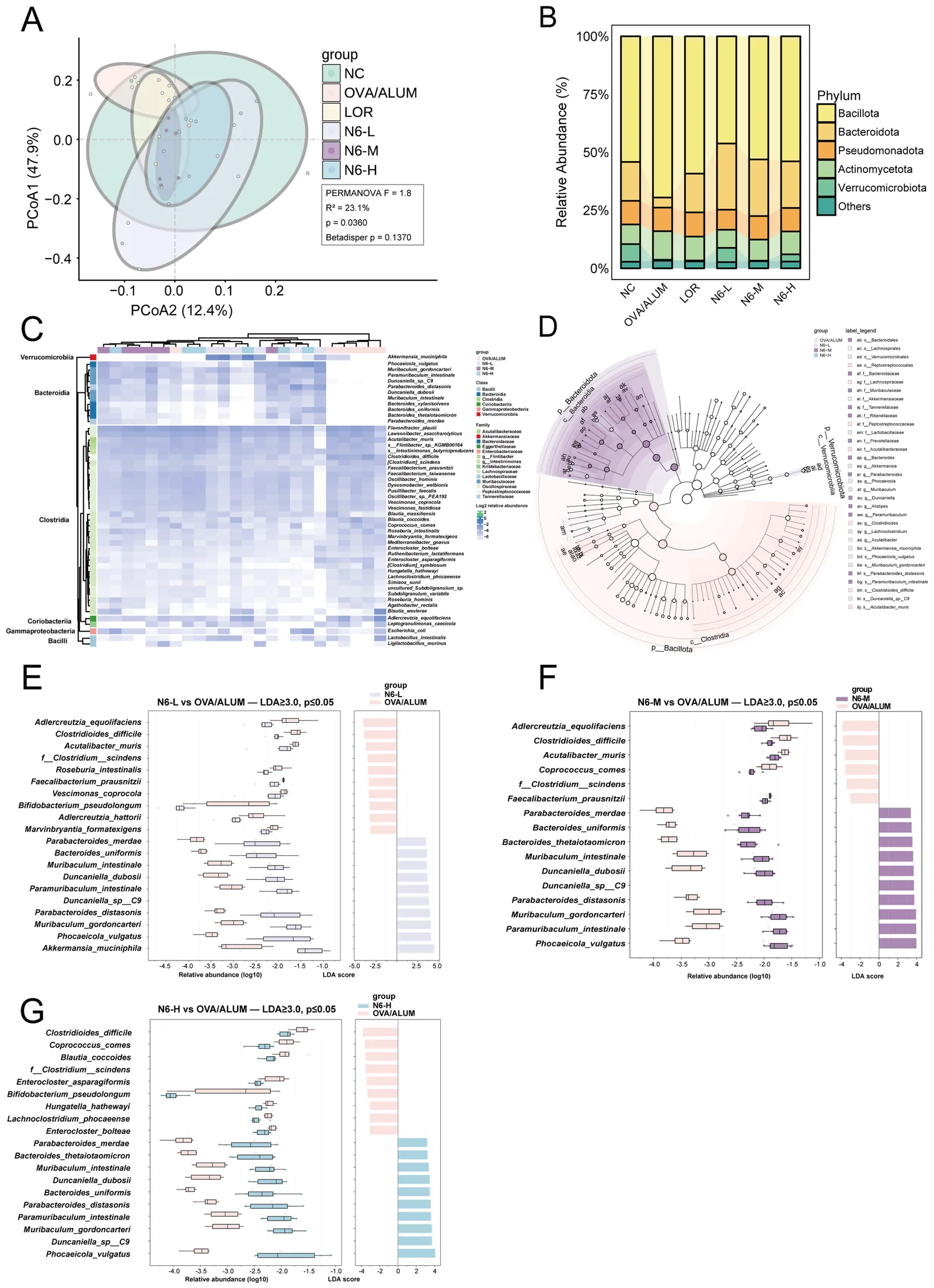
Effects of BGI-N6 on rat gut microbiota structure and composition. **(A)** Beta diversity of the rat gut microbiota, each dot corresponds to an individual animal (n = 6 per group). **(B)** Taxonomic profiles at the phylum level. **(C)** Hierarchical clustering heatmap of the top 50 most abundant species across the OVA/ALUM and BGI-N6 cohorts: Data are presented as log2-transformed relative abundances, clustered by microbial class (rows) and treatment (columns), with a color gradient (white-blue-green) indicating low to high abundance. **(D)** Linear discriminant analysis Effect Size (LEfSe) identifying key taxa enriched in the BGI-N6 groups versus the OVA/ALUM model (LDA score > 3.7, *p* < 0.05). **(E–G)** Dose-specific LEfSe evaluations at the species level comparing individual BGI-N6 groups against the disease model (LDA > 3.0, *p* < 0.05); Top ten species with the highest |LDA| scores in each group are presented. For these panels, log_10_-transformed relative abundances are plotted on the left, alongside the corresponding directional LDA scores on the right.

To explore the potential link between the immunomodulatory properties of BGI-N6 and gut microbiota restoration, metagenomic sequencing was performed to assess bacterial composition across groups. PCoA analysis revealed a distinct separation of the microbial community between the OVA/ALUM group and NC group. All BGI-N6-treated groups exhibited a composition shift toward the NC cluster, with the N6-H group showing the most pronounced convergence (**Figure 3**). At the phylum level, BGI-N6 administration significantly increased the relative abundance of Bacteroidota and decreased that of Bacillota compared with the OVA/ALUM group (**Figure 3**). Additionally, the abundance of Actinomycetota was significantly lower in the N6-L and N6-M groups, while Verrucomicrobia was notably enriched in the N6-L group.

A higher-resolution comparative analysis was next conducted between the BGI-N6-treated and OVA/ALUM groups to further resolve microbial compositional differences. The clustering heatmap of the top 50 species (**Figure 3**) identified Verrucomicrobiia, Bacilli, Clostridia, and Coriobacteriia as the dominant bacterial classes. Notably, the class Bacteroidia (phylum Bacteroidota) exhibited pronounced divergence between clusters, with significantly higher abundance in the BGI-N6-treated groups relative to the OVA/ALUM group. This clade encompasses 11 representative species, including the SCFA producers *Phocaeicola vulgatus* and *Bacteroides uniformis (*36) and the anti-inflammatory taxa *Parabacteroides distasonis* and *Parabacteroides merdae* (37, 38), *Muribaculum* and *Duncaniella*, members of the Muribaculaceae family, have been identified as core carbohydrate-degrading bacteria in the rodent gut (39). Conversely, the class Clostridia (phylum Bacillota) displayed an inverse pattern. The abundances of several species — including *Lachnoclostridium phocaeense*, *Simiaoa sunii*, *Subdoligranulum variabile*, *Roseburia hominis*, *Agathobacter rectalis*, and *Blautia obeum* — were elevated in the OVA/ALUM group. Although typically considered gut commensals, their aberrant enrichment in the AR model may be associated with allergic rhinitis.

LEfSe analysis was subsequently performed to statistically validate these compositional shifts, corroborating the heatmap findings with greater discriminatory power (**Figure 3D**; **Supplementary Figures 2A, B**). Notably, Clostridioides difficile was enriched in the OVA/ALUM group, indicative of gut dysbiosis and compromised colonization resistance. In contrast, BGI-N6 administration reduced the abundance of this potential pathogen while promoting the expansion of Bacteroidota-affiliated beneficial taxa, suggesting that BGI-N6 may alleviate AR-associated gut dysbiosis by enriching beneficial commensals and suppressing potentially harmful species. Additionally, *Akkermansia muciniphila* was significantly enriched in the N6-L group (40).

### 3.4 BGI-N6 modulates microbial functional pathways and core KOs associated with the alleviation of allergic inflammation

**Figure 4:**
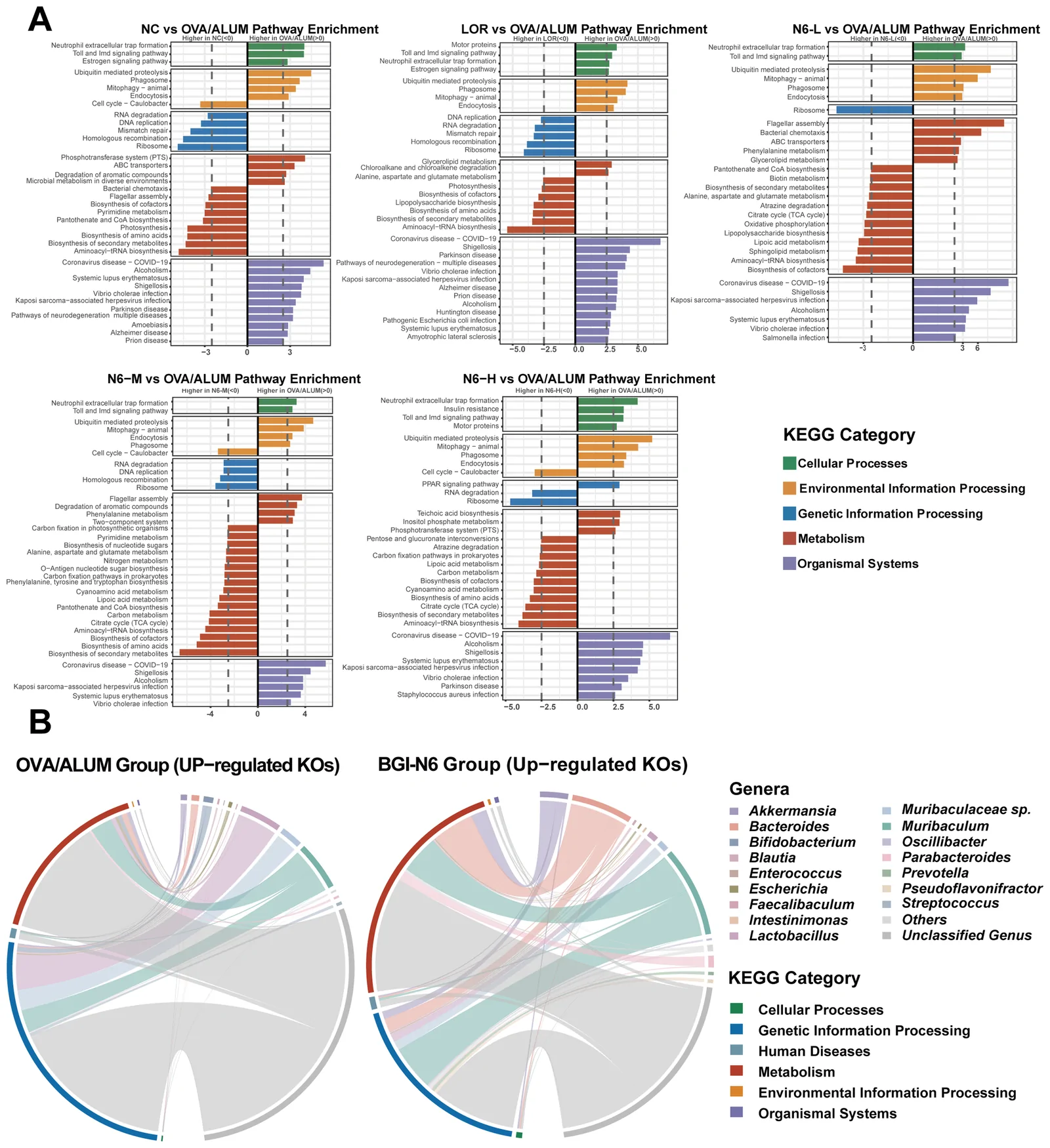
Effects of BGI-N6 on gene expression and metabolic pathways in AR rats. **(A)** Significantly activated and suppressed functional pathways across the different treatment and control groups versus the OVA/ALUM group (|Reporter Score| ≥ 2.5). **(B)** Chord diagrams showing genus-level microbial contributions to KEGG Level 1 functional categories to which consistently up-regulated (upper panels) core KOs in the OVA/ALUM and BGI-N6 groups. Arc lengths are proportional to relative abundance contributions, normalized to a global maximum across all panels. Wilcoxon rank-sum tests with Benjamini–Hochberg correction. FDR < 0.05.

Based on pairwise ReporterScore evaluations (**Figure 4**), the OVA/ALUM cohort exhibited elevated activity in pathways tied to innate immunity and cellular stress. This functional profile was characterized by the up-regulation of Environmental Information Processing networks—namely Phagosome, animal Mitophagy, Ubiquitin-mediated proteolysis, and Endocytosis—coupled with key Cellular Processes such as Neutrophil extracellular trap formation and Toll/Imd signaling. In contrast, BGI-N6-treated groups showed consistent enrichment in metabolic pathways, such as Biosynthesis of secondary metabolites, Citrate cycle (TCA cycle), Lipoic acid metabolism, and Aminoacyl−tRNA biosynthesis. The shift toward metabolic pathway enrichment suggests that BGI-N6 intervention may promote a metabolically active microbial community with enhanced capacity for producing immunomodulatory metabolites, potentially contributing to the restoration of gut immune homeostasis in AR. Among the three BGI-N6 groups, N6-M showed the greatest number of significantly enriched pathways.

To identify genes contributing to these functional shifts, we examined differential KO profiles across treatment groups. Compared to the OVA/ALUM group, differential KO analysis revealed distinct numbers of up- and down-regulated KOs across treatment groups: NC (205 up/52 down), LOR (195/10), N6-L (504/117), N6-M (243/40), and N6-H (174/50) (**Supplementary Figure 3A**). UpSet analysis of significant differential KOs across all dose groups identified 606 up-regulated and 173 down-regulated KOs in total, with the N6-L group containing the largest number of unique changes (**Supplementary Figure 3B**). To delineate the specific genetic KOs consistently associated with these pathway-level changes, we focused on 41 core KOs that exhibited strictly consistent regulatory trends across all three BGI-N6 dose groups. Taxonomic contribution analysis of the 41 core KOs (**Figure 4**) revealed a broader distribution of functional contributions across genera in the BGI-N6 groups compared to the OVA/ALUM group. Among these, *Akkermansia*, *Bacteroides*, and *Parabacteroides* showed higher functional contributions in the BGI-N6 groups, and several genera absent from the OVA/ALUM group, including *Intestinimonas*, *Oscillibacter*, and *Pseudoflavonifractor*, were newly detected in the BGI-N6 groups.

### 3.5 Correlations between gut microbial taxa, core KOs, and clinical phenotypic parameters in AR rats

**Figure 5:**
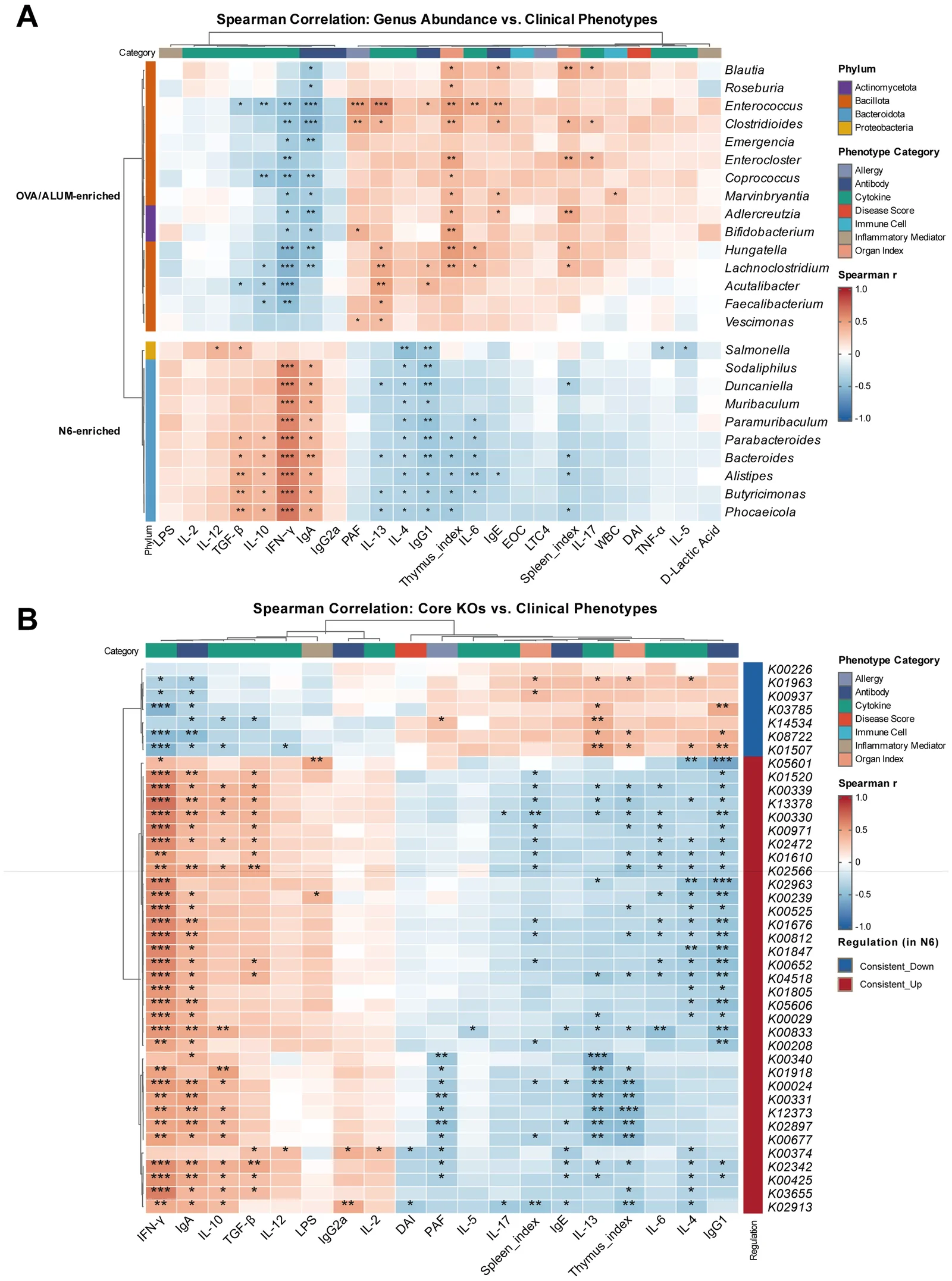
Spearman correlation analysis between gut microbial taxa, core functional genes, and clinical phenotypic indicators in AR rats. **(A)** Spearman correlation heatmap between gut bacterial genera and clinical phenotypic indicators. Genera were selected based on LEfSe significance (LDA ≥ 3.0, p < 0.05); all correlation p-values were Benjamini–Hochberg adjusted across the full genus–phenotype correlation matrix. Red, positive correlation; blue, negative correlation. Left bar, phylum; top bar, phenotype category. *p < 0.05, **p < 0.01, ***p < 0.001 (BH-adjusted). **(B)** Spearman correlation heatmap between core functional genes (KOs) and clinical phenotypic indicators. KOs were selected based on cross-dose consistency across the three BGI-N6 groups; all correlation p-values were Benjamini–Hochberg adjusted across the full KO–phenotype correlation matrix. Color scheme and significance thresholds as in Image. Right bar indicates regulation direction in BGI-N6 groups relative to OVA/ALUM (red, upregulated; blue, downregulated).

To elucidate the relationships among BGI-N6-induced microbial shifts, core KO alterations, and immune responses in AR rats, Spearman correlation analyses were conducted between the relative abundance of gut microbial genera and clinical phenotypic parameters (**Figure 5**), and between core KO abundances and clinical phenotypic parameters (**Figure 5**).

At the genus level (**Figure 5**), Bacteroidota-affiliated genera enriched after BGI-N6 administration, including *Parabacteroides*, *Bacteroides*, *Butyricimonas*, and *Phocaeicola*, showed positive correlations with Th1/Treg-associated anti-inflammatory markers (IFN-γ, IL-10, TGF-β, IgA) and negative correlations with pro-inflammatory and allergy-related indicators (IgG1, IL-4, IL-13, PAF). In contrast, Bacillota-affiliated genera enriched in the OVA/ALUM group, such as *Enterococcus*, *Clostridioides*, *Lachnoclostridium*, and *Hungatella*, displayed the opposite pattern. At the KO level (**Figure 5**), consistently up-regulated KOs were positively correlated with the same Th1/Treg-associated anti-inflammatory markers and negatively correlated with the same pro-inflammatory and allergy-related indicators, while consistently down-regulated KOs exhibited the inverse pattern. Spearman correlation analysis revealed that sphingolipid metabolism-related KOs(K12373) was positively correlated with IFN-γ, IgA, and IL-10, and negatively correlated with IL-13 and PAF, while aromatic amino acid biosynthesis-related KOs(K04518) was positively correlated with IFN-γ, IgA, and TGF-β, and negatively correlated with IL-4, IL-13, and IgG1. In contrast, down-regulated KOs including Oxidative phosphorylation-related KOs(K01507) and pyrimidine metabolism-related KOs(K08722) showed the opposite pattern. These convergent correlation patterns at both the genus and functional gene levels suggest that the compositional and functional shifts observed after BGI-N6 administration correlated with restoration of immune homeostasis in AR rats.

## 4 Discussion

The present study demonstrates that administration of *L. plantarum* BGI-N6 effectively alleviated AR symptoms and restored immune homeostasis in an OVA-induced rat model. These beneficial effects were accompanied by shifts in gut microbiota composition and microbial functional metabolic pathways. These findings extend the growing body of evidence that specific *L. plantarum* strains can modulate AR progression including immune regulation, gut microbiota optimization, intestinal barrier reinforcement, and attenuation of inflammatory cascades.

BGI-N6 administration significantly alleviated the hallmark AR symptoms — sneezing, nasal discharge, and nasal rubbing in AR rats, accompanied by visible improvement in nasal mucosal histopathology. These findings are consistent with previous reports of *L. plantarum* strains ameliorating allergic airway inflammation in rodent models (41, 42). Furthermore, BGI-N6 administration reduced peripheral WBC and EOS counts in AR rats. EOS are central effectors of allergic inflammation; their activation and degranulation lead to release of eosinophil cationic protein (ECP), which damage the epithelial barrier and perpetuates inflammatory cascades (43, 44). The reduction in circulating EOS counts observed here suggests that BGI-N6 may attenuate EOS driven mucosal injury, thereby preserving nasal epithelial integrity.

BGI-N6 administration not only significantly reduced serum levels of key allergic inflammatory mediators (IgE, LTC4, and PAF) but also elevated IgA levels. Elevated serum IgA levels are indicative of enhanced immune regulation, as monomeric serum IgA suppresses pro-inflammatory cytokine production and inhibits IgE-mediated mast cell degranulation, thereby contributing to immune homeostasis (45–48). Mechanistically, the pathogenesis of AR is fundamentally driven by dysregulation of CD4^+^ T cell differentiation, particularly Th1/Th2 imbalance and disruption of the Th17/Treg axis (49, 50). BGI-N6 counteracted these perturbations by suppressing the Th2-dominant response, reversing the elevated Th17/Treg ratio, and broadly rebalancing the pro- and anti-inflammatory cytokine network toward a more homeostatic state. Together, these changes collectively shifted the systemic immune milieu toward a more homeostatic immune state.

Beyond its immunomodulatory effects, BGI-N6 administration modulated the gut microbial composition in AR rats, with the shift toward a normal-like profile being most pronounced in the N6-H group. At the phylum level, the OVA/ALUM group showed increased Bacillota and decreased Bacteroidota abundance, a dysbiotic pattern commonly associated with allergic disease models. BGI-N6 administration reversed this imbalance, significantly increasing the relative abundance of Bacteroidota while decreasing that of Bacillota. LEfSe analysis further confirmed these shifts at a higher taxonomic resolution, revealing an enrichment of Bacillota-affiliated genera in the OVA/ALUM group, and of Bacteroidota-affiliated genera in the BGI-N6-treated groups. Notably, members of the Muribaculaceae family, including *Muribaculum* and *Duncaniella*, are core carbohydrate-degrading bacteria in the rodent gut. Their enrichment may reflect a restoration of fermentative capacity following BGI-N6 administration.

The enrichment of Bacteroidota-affiliated taxa may contribute to the immunomodulatory effects of BGI-N6 through the production of key microbial metabolites. Specifically, genera such as *Bacteroides*, *Phocaeicola*, and *Parabacteroides* are established producers of SCFA. SCFAs, particularly butyrate and propionate, are known to suppress Th2-driven allergic responses, promote Treg differentiation via histone deacetylase inhibition at the Foxp3 locus, and reinforce intestinal epithelial barrier integrity (51, 52). Furthermore, specific enriched species like *P. distasonis* are well-documented anti-inflammatory commensal that suppresses pro-inflammatory cytokine production, promotes Treg differentiation, and enhances intestinal barrier function across multiple inflammatory disease models (53, 54), consistent with the Treg expansion observed in the present study.

Functional metagenomic profiling revealed that BGI-N6 administration enriched pathways with direct relevance to immune regulation. The predominant enrichment of innate immune signaling and cellular stress pathways in OVA/ALUM groups reflects a state of gut microbial immune dysregulation under chronic allergen challenge. BGI-N6 intervention reversed this pattern, consistently shifting microbial functional capacity toward core metabolic pathways, including the TCA cycle, which provides key precursors for microbial SCFA biosynthesis, and biosynthesis of secondary metabolites. Taxonomic contribution analysis of core KOs suggested that BGI-N6 administration promoted the functional output of Bacteroidota-affiliated beneficial taxa, including *Bacteroides*, *Muribaculum*, and *Parasutterella*, within the gut microbial community.

The correlation analyses linked BGI-N6-induced microbial shifts to immune outcomes at both the compositional and functional levels. Bacteroidota-affiliated genera enriched after BGI-N6 administration showed consistent associations with anti-inflammatory markers, and the same directional patterns were observed for up-regulated core KOs, suggesting that compositional and functional changes in the microbiota occurred in a coordinated manner. Exploratory taxonomic contribution analysis, with unclassified reads excluded, further revealed that the core KOs most strongly associated with anti-inflammatory outcomes received increased contributions from Bacteroidota-affiliated taxa in BGI-N6-treated groups (**Supplementary Figure 4**). This suggests that BGI-N6 promoted not only the abundance but also the functional contributions of these beneficial taxa. Although the apparent dose optimum varied across individual metrics — most KOs altered at N6-L, most pathways enriched at N6-M, and greatest β-diversity convergence at N6-H — the overall response to BGI-N6 was directionally consistent. Clinical and immunological endpoints improved at all three doses (**Figures 1**, **2**), and metagenomic features significantly altered across all three dose groups showed consistent directional alignment across genera, pathways, and KOs (**Supplementary Table 2**). The metric-to-metric variation may reflect differences in dose sensitivity across analytical dimensions, differential statistical power, or inter-individual variability in baseline microbiota composition.

Several limitations should be noted. Colonization of BGI-N6 was not directly verified, and the absence of a heat-killed control leaves unresolved whether the observed effects depend on viable colonization or on paraprobiotic mechanisms. Microbiota profiling was confined to cecal contents, without nasal mucosal sampling or direct metabolite quantification. The microbiota–immune associations are correlational, the sample size (n = 6 per group) limits statistical power to resolve subtle dose-dependent effects, animals within each group were housed in a single cage such that cage and group effects cannot be separated, and only female rats were used, which may limit generalizability across sexes. Future studies incorporating both sexes, colonization tracking, direct SCFA quantification, matched nasal mucosal sampling, and lower airway evaluation would strengthen the mechanistic evidence for BGI-N6 in AR.

## 5 Conclusions

In conclusion, the present study provides evidence that oral administration of *L. plantarum* BGI-N6 is associated with alleviation of AR symptoms and improvement of immune homeostasis in an OVA-induced rat model, accompanied by enrichment of Bacteroidota-affiliated beneficial taxa and shifts in microbial functional output. These findings support further development of BGI-N6 as a candidate probiotic for AR. More broadly, they illustrate how multi-dose taxonomic and metagenomic profiling can provide a framework for linking strain-induced compositional shifts to predicted functional changes.

## Data Availability

The datasets presented in this study can be found in online repositories. The names of the repository/repositories and accession number(s) can be found below: https://db.cngb.org/data_resources/project/CNP0009371, CNP0009371.

## Supplementary material

Supplementary Table 1 Sensitivity analysis of LEfSe LDA thresholds (2.0–4.0, 0.1 increments) for all pairwise comparisons and the multi-group cladogram.

Supplementary Table 2 Directional concordance of features significantly altered across all three BGI-N6 dose groups, including concordant genera, pathways, and KOs with associated statistics. Serum cytokine levels across treatment groups (**Supplementary Figure 1**); species-level LEfSe enrichment analysis of NC and LOR groups (**Supplementary Figure 2**); differential KO volcano plots and UpSet analysis of BGI-N6 dose groups (**Supplementary Figure 3**); and genus-level taxonomic contributions to core KOs (**Supplementary Figure 4**).

Supplementary Figure 1 Serum cytokine levels across treatment groups in AR rats. **(A–K)** Serum levels of TNF-α, IL-4, IL-5, IL-6, IL-13, IL-17, IFN-γ, TGF-β, IL-2, IL-10, and IL-12, respectively. Data are presented as mean ± standard deviation (SD); *p < 0.05, **p < 0.01, ***p < 0.001, each dot corresponds to an individual animal (n = 6 per group).

Supplementary Figure 2 Species-level LEfSe enrichment analysis of NC and LOR groups relative to the OVA/ALUM group. Species-level LEfSe enrichment analysis comparing the NC group (A) and LOR group (B) with the OVA/ALUM group (LDA score > 3.0, p < 0.05). Top ten species with the highest |LDA| scores in each group are presented; the full list is provided in **Supplementary Table 1**. Left panel: log_10_-transformed relative abundance; right panel: LDA score (bar direction indicates enriched group).

Supplementary Figure 3 Differential KO analysis and genus-level functional contributions in the BGI-N6 allergic rhinitis model. **(A)** Volcano plot of differential KO analysis (DESeq2 with apeglm shrinkage) comparing each treatment group with OVA/ALUM. Red and blue points indicate significantly up- and down-regulated KOs (adjusted p < 0.05, |log_2_FC| ≥ 1); point size reflects –log_10_(adjusted p-value). Top 5 KOs per group by |log_2_FC| are labeled. **(B)** UpSet plots showing the intersections of differentially expressed KOs across BGI-N6 dose groups (N6-L, N6-M, and N6-H) relative to the OVA/ALUM group (adjusted p < 0.05). Upper panel: up-regulated KOs; lower panel: down-regulated KOs. Bar heights indicate the number of KOs in each intersection; filled circles and connecting lines denote the corresponding group combination. (C)Chord diagrams showing genus-level microbial contributions to KEGG Level 1 functional categories mediated by consistently up-regulated (down panels) core KOs in the OVA/ALUM and BGI-N6 groups. Arc lengths are proportional to relative abundance contributions, normalized to a global maximum across all panels. Wilcoxon rank-sum tests with Benjamini–Hochberg correction. *FDR < 0.05.

Supplementary Figure 4 Genus-level taxonomic contributions to consistently altered core KOs across treatment groups. Stacked bar plots showing the proportional contribution (%) of the top 9 genera to each core KO in the OVA/ALUM and BGI-N6 groups. Unclassified taxonomic entries were excluded prior to analysis. Abundances were summed across all samples within each group and normalized to 100%; remaining genera are aggregated as “Others”.
